# Primary healthcare managers’ perceptions of management competencies at different management levels in digital health services: secondary analysis

**DOI:** 10.1108/LHS-07-2022-0078

**Published:** 2022-10-11

**Authors:** Antti Ylitalo, Elina Laukka, Tarja Heponiemi, Outi Ilona Kanste

**Affiliations:** Research Unit of Nursing Science and Health Management, University of Oulu, Oulu, Finland and Health and Social Service System Research Group, Finnish Institute for Health and Welfare, Helsinki, Finland; Health and Social Service System Research Group, Finnish Institute for Health and Welfare, Helsinki, Finland; Research Unit of Nursing Science and Health Management, University of Oulu, Oulu, Finland and Medical Research Center, Oulu University Hospital, Oulu, Finland

**Keywords:** Health leadership competencies, Primary care, Management, Qualitative research, Management level, Digitalization, Primary health care, Management, Competence, Qualitative study

## Abstract

**Purpose:**

The purpose of this study is to describe primary health-care managers’ perceptions of management competencies at different management levels in digital health services using the management competency assessment program as a framework.

**Design/methodology/approach:**

A secondary analysis study involving 21 semi-structured individual interviews was conducted among Finnish primary health-care managers at different management levels (frontline, middle and senior). The deductive framework method was used to analyze the data.

**Findings:**

Similarities and differences were found in management competencies between different levels of management. Competencies related to the use of digitalization were highlighted by managers at all management levels. Managers at all management levels were involved in developing digital solutions and supporting employees in using digital solutions in their work. Frontline and middle managers emphasized more issues related to day-to-day management and communication with employees, whereas senior managers highlighted the management of large entities.

**Research limitations/implications:**

In the secondary analysis, data were used for purposes other than originally intended. Therefore, the data are subject to limitations of the methodology applied and should be transferred to other contexts with caution.

**Practical implications:**

Identifying the management competencies needed to manage digital health services is important to target managers’ training according to needs in the future.

**Social implications:**

The results could be used to develop the management of digital health services, as well as improve digital health services and their deployment.

**Originality/value:**

Previous literature mostly examined managers’ informatics competencies and paid little attention to other management competencies. This study discusses more broadly the management competencies that digital health services require from managers at different levels of management.

## Introduction

The health-care environment, which is increasingly focused on value-based purchasing, relies on performance and effectiveness and demands efficiency in patient care delivery (Hader, 2011). Digitalization in health care is one of the key solutions to increase effectiveness (Frennert, 2018). Health-care managers have a significant role in managing digital health services (Ingebrigtsen *et al.*, 2014; Lammintakanen *et al.*, 2010; Laukka *et al.*, 2020). In the past, managing clinical services was the main role of health-care managers (Sood *et al.*, 2017), but today they are increasingly responsible for the design, implementation and guidance of digital health services (Birken *et al.*, 2015; Laukka *et al.*, 2020; Sandström *et al.*, 2011). Managers at different levels of organizations play a vital role in organizational change (Maniam, 2012). Digital transformation represents one form of such organizational change, and it requires managers to have a variety of management competencies (Pihlainen *et al.*, 2016), such as informatics competencies, to successfully manage digital services (Collins *et al.*, 2017; Strudwick *et al.*, 2019). For example, it is important for health-care managers to set an example for employees as an active user of information technology (IT) (Lammintakanen *et al.*, 2010).

Competence includes skills, knowledge, attitudes, values and performance (Cowan *et al.*, 2005; Mikkonen *et al.*, 2018). Management competencies generally refer to the knowledge, skills and attitudes which managers need in their work and can be developed and learned (Katz, 1955; Yarbrough Landry *et al.*, 2012). Health-care management competencies are constantly changing (Pihlainen *et al.*, 2016). In this study, we examined management competencies in the context of digital health services, defined as the use of digital health or eHealth as digital technologies in health-related activities, including mobile-health (mHealth), telemedicine, telehealth, sensor-based monitoring, health IT and digital health games (WHO, 2020).

Previous research has found that health-care managers are often self-taught in using and managing digital health services (Collins *et al.*, 2017; Sharpp *et al.*, 2019). Different management levels require different competencies depending on whether the managers work in, for example, operational management or strategic management (Kantanen *et al.*, 2017). There appear to be differences in the management competencies of physician and nurse managers; e.g. often nurse managers receive better management training than physician managers (Herman *et al.*, 2015). Managers need to be provided with learning opportunities (Jennings *et al.*, 2007) that enable effective application of IT knowledge and skills in all management environments (Collins *et al.*, 2017; Remus and Kennedy, 2012; Simpson, 2004).

Health-care organizations traditionally have strong expertise that increases the hierarchy between different professional groups and supervisors (Vakkala and Syväjärvi, 2020). From the perspective of the challenges in managing a health-care organization, it is important that the managers responsible for it are qualified in many areas. Not all management levels require the same type of management competencies, but the competency requirements are determined by the management level (McGurk, 2010; Unal, 2017; Yarbrough Landry *et al.*, 2012). In health-care management, there are three main management levels: frontline, middle level and senior level (Adindu and Asuquo, 2013). Low- and high-level managers differ in many ways and are expected to do different things. For example, employees need more interpersonal traits in low-level managers than high-level managers and more dominant traits in high-level managers than low-level managers (Nichols and Cottrell, 2014).

Ingebrigtsen *et al.* (2014) reported that clinical managers need to develop their IT skills, build partnerships with IT professionals and implement proactive IT behavior to succeed in deploying digital services. IT skills and knowledge play a vital role in the management and development of digital health services. They also noted that managers with previous experience in project management as well as health-care technical expertise are likely to develop a vision that includes a long-term commitment to the use and development of IT tools. Similar thoughts were expressed by Collins *et al.* (2017), who identified 74 competencies related to IT that were relevant to and practiced by nursing managers.

Strudwick *et al.* (2019) suggested 11 competency themes relating to managers’ informatics skills, informatics knowledge and other competencies, e.g. computer skills. The identified competencies included ones specific to technology and information systems and broader competencies that nurse managers require to be effective in their roles. For example, broader competencies included quality management and operations skills. While skills and knowledge of IT are important, other competencies are also needed to manage digital health services. Previous studies have mostly scrutinized informatics competencies (Collins *et al.*, 2017; Strudwick *et al.*, 2019) and paid little attention to other management competencies, such as those related to leading employees in a digital environment. The present study examined more broadly the management competencies required from managers in the context of digital health services. The study also compared management competencies between different levels of management.

In this study, the management competency assessment program (MCAP) was used as a framework (Liang *et al.*, 2018) because it is the most recent and validated framework for health service managers and includes behavioral items that can measure each of the core management competencies (Liang *et al.*, 2018). The MCAP contains six core competencies: evidence, resources, knowledge, leadership, communications and change. Evidence competence requires evidence-based decision-making. Resources include administration, operations and resource management. Knowledge includes knowledge of the organization and the operating environment of health care. Communications competence concerns interpersonal relationships, communication capabilities and relationship management. Leadership includes leading people and the organization. Change includes enabling, managing and related features of change (Liang *et al.*, 2018).

The aim of this study was to describe primary health-care managers’ perceptions of management competencies (evidence, resources, knowledge, leadership, communications and change) in digital health services at different management levels using the MCAP as a framework. The main research question was:

RQ1. What perceptions do primary health-care managers have about management competencies in digital health services at different levels of management?

## Methods

### Study design

In this study, secondary analysis was used. In secondary analysis, data are used for a purpose other than originally intended (Long-Sutehall *et al.*, 2011). Secondary analysis of qualitative data was conducted by using a preexisting interview data set.

### Study settings

This study focused on primary health care and the managers working therein. In Finland, primary health care comprises the key functions of health care, which are generally available to everyone and form the core of the country’s health system (Ministry of Social Affairs and Health, 2019). Four primary health-care centers from different parts of Finland participated in the study. These centers were chosen because they all had a long background in providing digital health services. For example, they all used several different digital health services, such as electronic health records, knowledge management support systems, patient portals and symptom checkers.

### Participants and data collection

The material for the secondary analysis study was collected through semi-structured interviews in May and November 2020 from 21 managers of primary health care from different parts of Finland, i.e. three municipalities and one association of municipality. A semi-structured interview method was used to collect the data because the perceptions of the managers were of interest. Purposive sampling (Tongco, 2007) was used to recruit health-care managers. The inclusion criteria for participation were as follows:

- having sufficient knowledge/understanding of digitalization;
- being able to represent the organization from a policy or guideline perspective; and
- working as a frontline manager, middle manager or senior manager in primary health care.

Appropriate managers were contacted and informed of the study by e-mail.

A total of 21 managers agreed to participate. Most managers were female (*n* = 19), and the majority (*n* = 18) had a clinical background: 12 with nursing and 6 with a medical background. The age of the interviewees varied between 35 and 65 years (average 50 years), and they had 1 to 20 years of management experience (average 8 years). Managers worked as frontline managers (*n* = 9), middle managers (*n* = 8) and senior managers (*n* = 4).

The interviews were conducted using Microsoft Teams, except for one interview, which was conducted over the telephone due to connectivity issues. One researcher (*[initials of author removed for peer-review]*) conducted the interviews. Interviews were recorded and later transcribed word for word, and the transcripts were anonymized. Interviews were continued until saturation was reached; i.e. responses of the same content emerged from the interviews. The interviews lasted for 25–60 min and generated a total of 850 min of data, resulting in a total of 232 pages of transcribed material (Verdana, font size 11, line spacing 1.15).

### Ethical considerations

Research Ethics Committee approval from the Finnish Institute for Health and Welfare was obtained (THL/2304/6.02.01/2020), and research permission was granted by each participating organization. In addition, interviewees were asked for written informed consent. A bulletin was sent to all interviewees mentioning the voluntary participation of the study and the right to suspend participation at any time. In addition, the interviewees had the opportunity to read a privacy statement. The interviews were recorded only after permission was obtained from the interviewees, and the recordings were only available to the researchers. The results were reported in such a way that individual interviewees could not be identified.

### Data analysis

The data were analyzed using the deductive framework method (Gale *et al.*, 2013). In a deductive analysis, an unstructured analysis framework is based on previous information, in this case the MCAP framework (Liang *et al.*, 2018). Initially, the transcribed material was read through several times to obtain an overview. The coding framework used was deductive, i.e. the material was coded for matters relevant to our research design. Inductive generated open codes were also included to complement the deductive analysis. All management levels were analyzed separately and compared. We identified 102 codes for frontline managers, 81 for middle managers and 37 for senior managers, giving a total of 220 codes. Next, codes that were similar in content were formed into categories. Finally, all categories were recorded in a framework matrix based on the MCAP. The MCAP contained a total of six core competencies under which categories were formed. Three (*[initials of author removed for peer-review]*) researchers participated in the analysis of the material, identified similarities and differences in their analyses and compiled a final analysis.

### Rigor

The trustworthiness of the study was assessed by the criteria of credibility, dependability, transferability and confirmability (Lincoln and Guba, 1985). The credibility was improved by selecting appropriate interviewees according to the inclusion criteria. The dependability was increased by the participation of three researchers in the data analysis, which allowed revision of the classification logic. Efforts were made to improve the confirmability by clearly recording the progress of the research process. The use of direct quotations supported the authenticity of the results. The transferability of results was improved by interviewing managers belonging to different management levels in different organizations and by clearly describing the background information of the interviewees.

## Findings

The analysis of the data focused on management competencies according to the MCAP, including evidence, resources, knowledge, communications, leadership and change (Table 1). A total of 51 categories were formed in the analysis phase, of which 20 concerned frontline managers, 18 middle managers and 13 senior managers.

### Evidence

Evidence included two categories: IT and digitalization skills and managing with knowledge. The main competence was IT and digitalization skills, which were emphasized at all levels of management. According to the results, managers needed digital skills to lead digital health services. Insufficient IT skills of managers made them feel uncertain and concerned. As the managers felt responsible for the management of IT services, they needed IT skills themselves. They also needed to be able to constantly maintain and learn new IT skills, such as use of new electronic services and digital systems. Managers thought that they had to learn new things themselves to teach employees who needed support in using the technology. Managers improved their IT skills by self-training but occasionally obtained their IT skills from training:

I think the manager should have the digitalization skills as well. (Middle manager 8)

Well, of course, it is required that you know how to use these digital systems […] (Frontline manager 2).

The other category was managing with knowledge, which was particularly emphasized in middle and senior management levels. Managers had to have sufficient digital knowledge and needed to be able to take advantage of digital data to lead with knowledge. Their aim was to make their decisions based on knowledge. Managing and using digital information has become part of the day-to-day operations of health-care organizations and work communities. The health-care industry and management of digitalization are, in the views of the managers, strongly based on knowledge and its use:

[…] we need to have up-to-date knowledge management. (Middle manager 3)

### Resources

Resources included six categories: awareness of what is happening in the organization, planning digitalization, digital service process design, coordination activities, investing in digital solutions and deployment of digital solutions. Frontline and middle managers highlighted that they needed to be aware of what was happening with digitalization in their organization; i.e. they needed to be aware of digitalization developments and activities taking place in the organization. This allowed them to communicate new things to employees and develop their own workplace operations concerning the digitalization of health care. At the same time, they were able to develop their own knowledge of digitalization according to the needs of the organization:

[…] I have to know what is going on in here [in the organization] […] (Frontline manager 1).

Frontline and senior managers perceived that planning digitalization was one of the competencies needed when managing digital services. The digitalization of health care requires new methods and systems for the workplace. The coronavirus pandemic has rapidly changed the digital work culture in health care, and the digital transformation is seen to be permanent. The managers had to participate in the development of electronic services and support the development work. They also wanted to ensure sufficient resources for development. They needed competence in planning to detect the possibilities of digital solutions and the ability to deploy digital services. Based on managers’ perceptions, listening to employees’ worries and wishes during the planning was essential.

According to frontline and senior managers, the design of service processes was an important competence. They wanted to redesign digital service processes with digital tools and services. The middle managers emphasized the coordination of activities. They felt that they needed to be aware of what was going on in the workplace and to be able to coordinate it. Only senior managers highlighted that investment in digital equipment and solutions was one of the competencies they needed. Senior managers also highlighted the deployment of digital devices. In their view, they needed to use resources to invest in new digital systems and equipment, and they needed to ensure that employees also knew how to use new solutions:

[…] requires the design of these service processes on a completely new basis […] (Senior manager 2).

### Knowledge

Knowledge included two categories: knowledge of digitalization and utilization of different perspectives. Knowledge of digitalization was considered an important competence at all levels of management. However, some managers felt that they did not have sufficient knowledge about digitalization. Managers at all levels highlighted that they should have adequate knowledge of digital solutions and programs so that they knew how to use them. Understanding digitalization increased knowledge of the opportunities it enabled for the organizations. Managers also perceived that they were able to manage and make better use of information through digital solutions. Frontline managers also emphasized the value of looking at things from different perspectives, such as from the perspectives of customers, management and the work community. They thought it was important to take different perspectives into account when making decisions:

[…] organizational managers understand that health care and all other services are rapidly digitalizing […] (Senior manager 2).

[…] think about the customer, the work community, the management, and the finances from every aspect […] before making a decision […] (Frontline manager 6).

### Communication

Communication included five categories: listening to employees, empathy for employees, cooperation with technical professionals and developers, marketing of digitalization for employees and creating networks. Frontline and middle managers emphasized competencies related to digital communication with employees. This is understandable because frontline and middle managers work with staff more than senior managers. For example, they wanted to listen and be empathetic to employees because the digitalization of health care is undergoing a lot of change and the work is often stressful. Remote work posed challenges to meeting and supporting employees because managers were not able to deal as much with employees as before. Frontline managers felt that they needed to be able to maintain social relationships with employees as well as maintain group dynamics in the workplace. Managers had to find ways in which they could detect problems in the work community. Remote working may also lead to managers and employees not meeting physically as much in the workplace. Managers must be able to ensure good communication and find ways to listen to employees even in changing circumstances:

[…] listening to employees as to what aspirations they have or what jobs are not going well. (Frontline manager 4)

Frontline managers expressed that they needed to collaborate with technical professionals and developers. They emphasized the importance of communication with technical professionals to enable operations to proceed as smoothly as possible. They thought that they needed communication and support from technical professionals because frontline managers were often responsible for the deployment of digital health services. The supportive relationship between frontline managers and technical professionals was bidirectional. Frontline managers felt they also needed to provide support to technical developers and needed to communicate with them. They needed to be able to develop digital health services together with developers. Frontline managers also delivered ideas from employees to the developers based on employees’ experience of how systems worked in everyday practical work:

[…] we need strong collaboration and support and discussion with technical and digital professionals. (Frontline manager 3)

Frontline managers emphasized the marketing of digitalization to employees and evaluation of operations. They perceived that they needed to explain the opportunities of digitalization to their employees. For this reason, managers needed to stay abreast of the opportunities that digitalization brings. In addition, middle managers stressed the importance of networking, e.g. by collaborating with other professional groups:

[…] what kind of service you plan to market to your employees […] (Frontline manager 8).

### Leadership

Leadership included four categories: supporting employees, ensuring employees’ training, motivating employees and performance evaluation. Managers at all management levels highlighted the varying IT skills of employees. Managers raised concerns about the insufficient IT skills of older workers but stated that younger workers usually had good IT skills and were generally happy to help older employees with the use of IT. Managers at all management levels highlighted the need to support employees. Leading digital change involved encouraging employees to keep up with the change and providing support for new ways of working digitally. Managers also had to be able to ensure employees’ training concerning the use of various digital services. Some managers said they were training their employees themselves or trying to ensure employees had access to training. Frontline and middle managers emphasized motivating employees in their perceptions. Managers must be able to motivate their employees to use digitalization and learn new things. In addition, frontline managers highlighted that they needed to be able to evaluate an organization’s performance, e.g. using statistics:

Managers are supporters, encouragers, enablers […] (Frontline manager 8).

We need to take care of training, orienting and motivating our employees. (Middle manager 3)

### Change

Change included seven categories: development of digitalization, being excited by digitalization, having a positive attitude toward digitalization, learning digitalization, updating digital information, innovative thinking and visionary thinking. Managers at all management levels expressed that they want to be involved in developing the digitalization of health care. Managers at all management levels highlighted being excited by digitalization and a positive attitude toward digitalization. Managers’ perceptions of attitude seemed to be related to how they felt about digital services. For example, a positive attitude has a positive effect on the desire to learn and embrace new things. Managers needed to be familiar with digital solutions and to see digitalization as an opportunity in health care. Managers perceived that the attitudes of managers also affected the attitudes of employees. A positive attitude was important at all levels of management:

And having a positive attitude towards the digitalization. Even in this situation, the attitude is key. (Frontline manager 7)

Frontline and middle managers also perceived that they needed to maintain their knowledge and anticipate the future. New digital solutions are constantly being added to health care. Managers needed to keep up with developments and update their knowledge. They also wanted to find new ways and methods of working with digital solutions. To do this, they needed information about changes underway. Frontline and middle managers also emphasized information retrieval skills and learning new things:

[…] yes, it takes knowledge of this [digitalization], and one must stay up to date and train oneself and somehow keep up with what there is to offer [digital solutions]. (Middle manager 1)

Middle managers also emphasized innovative thinking. They needed to be able to innovate with new solutions as well as boldly implement them. Senior managers perceived that they had to be visionary managers. They needed new ways of working and digital solutions for service systems. In addition, they needed so-called out-of-the-box thinking competency:

Managers must be visionary and commit to the development and deployment of digital services. (Senior manager 1)

## Discussion

This study was based on the perceptions of Finnish primary health-care managers about management competencies at different management levels in digital health services. We identified different management competencies that health-care managers need in managing digital health services. Similarities and differences were found in the competencies between different management levels. Competencies related to the use of digitalization were highlighted at all levels of management. In addition, managers at all levels needed to be involved in developing digital solutions and supporting employees in using digital solutions. Frontline and middle managers emphasized more issues related to day-to-day management and communication with employees, whereas senior managers highlighted the management of large entities.

According to our results, managers need to be able to justify their decisions based on knowledge. Thus, our results emphasize knowledge management, which is congruent with a previous study (Kakemam *et al.*, 2020). In accordance to our study, previous research has found that managers need to have adequate IT skills (Collins *et al.*, 2017; Ingebrigtsen *et al.*, 2014; Strudwick *et al.*, 2019). Based on our study, insufficient IT knowledge causes uncertainty and concern in managers. A previous study showed that some health-care managers need more training and support to lead digital health services (Laukka *et al.*, 2020). In our study, managers were also concerned about employees’ IT skills. Managers first need to learn new IT-related knowledge and skills themselves so that they can justify their decisions and teach employees who needed support in using the technology.

Based on our study, managers need to be aware of what is going on in the organization, participate in the development of digital services and set aside sufficient resources for these. Awareness of what is happening in the organization is important so that managers can identify needs where digital solutions can be leveraged. Previous research has shown that managers need to be involved in various activities at different organizational levels (Pihlainen *et al.*, 2016; Strudwick *et al.*, 2019) and participate in the development of digital services (Laukka *et al.*, 2020; Sandström *et al.*, 2011). For example, Strudwick *et al.* (2019) noted that health-care managers need to be involved in decision-making related to the strategic direction, acquisition, selection, deployment, use and evaluation of technologies.

According to our results, various aspects of knowledge emerged in the perceptions of managers. Other studies have also found that managers need to have sufficient knowledge of digital solutions (Collins *et al.*, 2017; Ingebrigtsen *et al.*, 2014; Laukka *et al.*, 2020; Strudwick *et al.*, 2019). Managers also need to be able to maintain their IT-related knowledge and acquire new knowledge. To lead digital health services, managers need to have sufficient knowledge of digital health services and their potential. Managers also felt they were better able to manage information with digital solutions. The importance of knowledge and its use in the management of bigger entities has been emphasized in a previous study (Pihlainen *et al.*, 2016).

In our study, we found that the coronavirus pandemic caused a rapid digital shift in health-care organizations, which changed the work culture of the organizations. Previous research has found that managers are concerned about the technological skills of their employees (Lammintakanen *et al.*, 2010). Managers must strive to identify the different skills needs and strengths of their employees. This is important so that more emphasis can be placed on technological training for employees. Our results revealed that managers must strive to communicate with employees and the rest of the work community. For example, they need to enable training and support and motivate employees to use of digital services.

We found that frontline managers emphasized collaboration with technical professionals and developers. Frontline managers wanted their and their employees’ ideas to be considered in the preparation and design of digital solutions. They also felt that they needed to obtain sufficient guidance and perspectives from a technical professional. Managers perceived that co-operation was necessary to make services smooth and to reap the full benefits of digitalization. Similar findings have been reported in previous studies. For example, a review by Ingebrigtsen *et al.* (2014) concluded that health-care managers need to work with IT professionals to develop and make digital solutions work.

In our study, managers highlighted the benefits of a positive attitude and excitement for digitalization. The positive attitude of managers affected the entire work community and organization. In addition, a positive atmosphere improved managers’ and employees’ attitudes toward digitalization. Managers emphasized the importance of an excited mindset to develop digital services and find new service solutions for work communities and organizations. For example, managers wanted to be involved in the development of e-services and support development work. As digital solutions increase, managers will play a significant role in service development (Ingebrigtsen *et al.*, 2014). Managers’ attitudes have an impact on the management and use of digital solutions (Ingebrigtsen *et al.*, 2014; Lammintakanen *et al.*, 2010).

One of the aims of this study was to examine differences in management competencies between different levels of management. Previous studies have identified the need for a review between management levels in management competencies (Kakemam *et al.*, 2020; Liang *et al.*, 2018). This study brings novelty value to this topic. The content of competencies varied somewhat between management levels. The perceptions of frontline and middle managers included more competencies related to leading people and communicating with employees, whereas senior managers emphasized competencies related to planning and investing. However, senior managers did not express communication-related competencies. This might be because the work of senior managers does not involve as much communication with employees as frontline and middle managers do.

Frontline managers and middle managers seemed to place more emphasis on leading smaller entities and communicating with employees, whereas senior managers highlighted the importance of managing larger entities. Our results confirmed previous studies related to the review of management levels (McGurk, 2010; Nichols and Cottrell, 2014; Unal, 2017; Yarbrough Landry *et al.*, 2012). For example, Unal (2017) noted that core competencies may vary significantly between different management levels depending on the sector, position and level of management of the manager. Identifying competency needs and providing training to tackle competency gaps on an individual basis could raise the performance level of management. This would also help to develop the competencies of health-care managers.

## Conclusion

Overall, the MCAP served as a good framework for identifying management competencies between different levels of management. Based on our study, MCAP may also be used in the context of digital health services. This study brings new knowledge and provides a broad perspective on management competencies at different management levels in digital health services. The study provides new evidence and promotes management competencies in research. Competencies related to the use of digitalization were highlighted at all levels of management. Senior managers seemed to emphasize the management of larger entities, whereas frontline and middle managers had more leadership in smaller entities.

It is important to invest in the training and development of managers to increase the competencies of managers in digital health services. The results of this study could help steer the training and education of health-care managers to better support their digitalization competence. As digitalization becomes more prevalent in health care, managers need to master new skills to incorporate digitalization smoothly in their day-to-day work. In the future, it would be necessary to develop an instrument for measuring the management competencies in digital health services to study effectiveness of training interventions. In addition, research on the management competencies should be done continuously, as digitalization is constantly shaping the field of health care.

## Limitations

The transferability of the results is limited by the focus of the research on the context of primary health care. The research results may, where applicable, be transferable to the corresponding primary health-care units. The questions asked in the interviews were not specified for the research question of this secondary analysis study, which may undermine the dependability of the study. The credibility of the study may be impaired by the fact that the interviews were not guided by the research question in this study. Gender distribution may also have affected the results of the study.

## Figures and Tables

**Table 1.** Management competencies at different management levels in digital health services according to the MCAP

Management competencies	Frontline manager	Middle manager	Senior manager
Evidence – evidence-informed decision-making	IT and digitalization skills	IT and digitalization skills managing with knowledge	IT and digitalization skills managing with knowledge
Resources – operations, administration and resource management	Awareness of what is happening in the organization Planning digitalization Digital service process design	Awareness of what is happening in the organization Coordination activities	Planning digitalization Digital service process design Investing in digital solutions Deployment of digital solutions
Knowledge – knowledge of health-care environment and the organization	Knowledge of digitalization Utilization of different perspectives	Knowledge of digitalization	Knowledge of digitalization
Communications – interpersonal, communication qualities and relationship management	Listening to employees Empathy for employees Cooperation with technical professionals and developers Marketing of digitalization for employees	Listening to employees Empathy for employees Creating networks	
Leadership – leading people and organization	Supporting employees Ensuring employees’ training Motivating employees Performance evaluation	Supporting employees Ensuring employees’ training Motivating employees	Supporting employees Ensuring employees’ training
Change – enabling and managing change	Development of digitalization Being excited by digitalization Having a positive attitude toward digitalization Learning digitalization Updating digital information	Development of digitalization Being excited by digitalization Having a positive attitude toward digitalization Learning digitalization Updating digital information Innovative thinking	Development of digitalization Being excited by digitalization Having a positive attitude toward digitalization Visionary thinking
